# Understanding the complexity of living with, and managing, secretions in motor neuron disease/amyotrophic lateral sclerosis (MND/ALS/ALS): protocol for a complex intervention systematic review

**DOI:** 10.1136/bmjopen-2025-103704

**Published:** 2025-10-10

**Authors:** Caroline Barry, M Farquhar, Matthew Hawkes, Charlotte Massey, Jane L Cross

**Affiliations:** 1 Norfolk and Norwich University Hospitals NHS Foundation Trust, Norwich, UK; 2 University of East Anglia, Norwich, UK; 3 Department of Neuroscience, The University of Sheffield, Sheffield, UK

**Keywords:** Motor neurone disease, PALLIATIVE CARE, Respiratory Therapy, Systematic Review, Quality of Life

## Abstract

**Abstract:**

**Introduction:**

Motor neuron disease/amyotrophic lateral sclerosis (MND/ALS/ALS) is an incurable disease which leads to muscle weakness that worsens over time. MND/ALS is highly heterogeneous in its presentation, with many people experiencing a rapidly progressive trajectory of symptoms. Many people living with MND/ALS (plwMND/ALS) experience a combination of flaccidity and spasticity of the muscles involved in speech, swallowing, breathing and coughing. This makes it challenging to deal with the saliva and mucous (‘secretions”) produced by the body. Failure to manage these problems effectively can lead to accumulation and aspiration of secretions, which may cause pneumonia and respiratory insufficiency. Knowing the best way to treat this problem is a challenge. Systematic reviews report substantive ongoing uncertainty regarding secretions management (SM). Little is known about the comparative effectiveness of secretion management interventions, their impact on quality of life and acceptability for plwMND/ALS and their unpaid/family.

**Methods and analysis:**

A complex intervention systematic review of SM for plwMND/ALS and/or their carers will be conducted using an iterative logic model approach, designed in accordance with the principles and guidance laid out in a series of articles published by the Agency for Healthcare Research and Quality on complex intervention reviews . Eight electronic databases will be searched for publications between 1996 and present: Ovid Embase, EBSCO CINAHL, EBSCO Academic Search Ultimate, Scopus, EBSCO PsycInfo, Ovid MEDLINE and the Social Sciences Citation Index. This will be supplemented by hand searching of reference lists of included studies. Two reviewers will independently screen the results for potentially eligible studies using AS Review Lab (a semi-automated machine learning tool). Study selection, data extraction and risk of bias assessment, using Gough’s Weight of Evidence Framework, will be independently performed by two reviewers. A framework thematic synthesis approach will be employed to analyse and report quantitative and qualitative data. The reporting will be conducted in line with the Preferred Reporting Items for Systematic Review and Meta-Analysis Complex Intervention Extension Statement and Checklist.

**Ethics and dissemination:**

This review will involve the secondary analysis of published information; therefore, ethical approvals are not required. Dissemination will be via presentation at scientific meetings, presentations to MND/ALS support groups and publications in peer-reviewed journals.

**PROSPERO registration number:**

CRD42025102364.

**STRENGTHS AND LIMITATIONS OF THIS STUDY**

- This will be the first complex intervention systematic review on secretion management (SM) in motor neuron disease/amyotrophic lateral sclerosis (MND/ALS), offering a comprehensive assessment of its effectiveness and impact.
- Logic modelling will illustrate the complexity of SM interventions for people with MND/ALS, their carers and healthcare professionals, aiming to inform policy and practice.
- Input from people with lived experience and MND/ALS specialists will enhance the relevance of the findings.
- The review may be limited by a lack of empirical evidence and variability in study designs, populations, outcomes and interventions.
- Restricting the review to English-language studies may limit generalisability across different settings and health systems.

## Introduction

 Motor neuron disease/amyotrophic lateral sclerosis (MND/ALS) is a progressive, neurodegenerative condition, with a prognosis of around 2–4 years from diagnosis. The location of onset of muscle weakness varies from limbs, speech and swallow (known as bulbar) or respiratory muscles. There are limited therapeutic options in terms of halting or slowing disease progression, and no cure. Current optimal clinical management is facilitated by multidisciplinary team (MDT) care, with a focus on relief of the difficult symptoms associated with MND/ALS.

Secretions management (SM) is a complex aspect of MND/ALS care, with around 42% of people living with MND/ALS (plwMND/ALS) affected by this problem. Secretions are multifactorial in nature and have a major impact on plwMND/ALS and their family carers, with symptoms that are distressing to experience and witness.^1^ Symptoms are often poorly managed and, in contrast to other elements of the MND/ALS pathway, the optimal care approach is poorly defined, with multiple healthcare professionals involved in managing the problem.^2^

Here, ‘secretions’ refers to fluids arising from either the oral cavity (namely saliva/sialorrhoea), respiratory system (mucus) or both. Oral and respiratory secretions may be thick, thin or mixed.^3^ Sialorrhoea refers to excessive salivation and/or drooling and is most commonly due to bulbar dysfunction (weakness of pharyngeal muscles and reduced swallowing capacity) and affects up to half of plwMND/ALS.^4^

There are several pharmacological and non-pharmacological interventions for secretions management, however evidence to support their use is sparse. A recent Cochrane review focusing on the management of excessive saliva (not respiratory secretions) found evidence of low-moderate certainty for two drugs used for this problem: botulinum toxin B (Botox) injections and oral dextromethorphan with quinidine (DMQ).^2^ Neither is considered first-line treatment: access to Botox injections is variable across the UK and DMQ is not currently licensed for use in the UK.

National Institute for Health and Care Excellence (NICE) Guidance recommends the use of anti-muscarinic medication, and botulinum toxin A for sialorrhoea, in addition to non-pharmacological measures such as advice on swallowing, diet, posture, oral care and suctioning for the management of thin saliva.^5^ Where thick saliva is present, NICE Guidance suggests a range of non-pharmacological measures, humidification, nebulisers and carbocysteine. No interventions are recommended for a mixed picture, where both thick and thin secretions exist, despite the high prevalence of this issue in clinical practice.^3^ Treatment failure rates are high, with evidence that certain interventions (such as anti-muscarinic) may worsen symptom management and/or be associated with intolerable side effects.^3^

Interventions used to manage secretions in MND/ALS (both pharmacological and non-pharmacological) may be influenced by a variety of individual, contextual and environmental factors, such as the extent of physical limitations, presence of a carer (a paid carer or unpaid/family carer), access to respiratory physiotherapy and/or palliative care services, use of non-invasive ventilation and care setting.

SM can be understood as a complex intervention, where the nature of the interventions themselves (such as the number of components involved, or expertise required) or through interactions between the intervention and its context are complex.^6^

This review aims to understand how the key components of SM interventions lead to beneficial effects, and in what context. It will articulate the key components of SM interventions, how they interact, the mechanisms of the interactions and how these might vary by context.^6^

The review questions are:

1. What secretion management interventions work, for whom and in what circumstances? (population, intervention, setting).
2. What are the mechanisms by which secretion management interventions are implemented by plwMND/ALS, their carers and care professionals (population, intervention, context)?
3. What is the impact of secretions, and/or their management interventions on plwMND/ALS and their carers? (outcomes).

## Methodology

### Approach

Identifying SM as a complex intervention requires identification of mechanisms of change, important contextual factors and relevant outcome measures.^6^ Complex intervention systematic reviews seek clarity on how such externalities influence outcomes in a population and are therefore considered more appropriate to our research questions than a conventional systematic review focusing on efficacy.

Conducting a systematic review of SM interventions requires an iterative approach to fully explore the complexity before determining the exact focus of the review.^7^ Logic models can add value and impact to systematic reviews by highlighting underlying assumptions about causal relationships, promoting systems thinking and identifying aspects of complex problems to decision makers in a more transparent and cogent way.^8^

**Figure 1:**
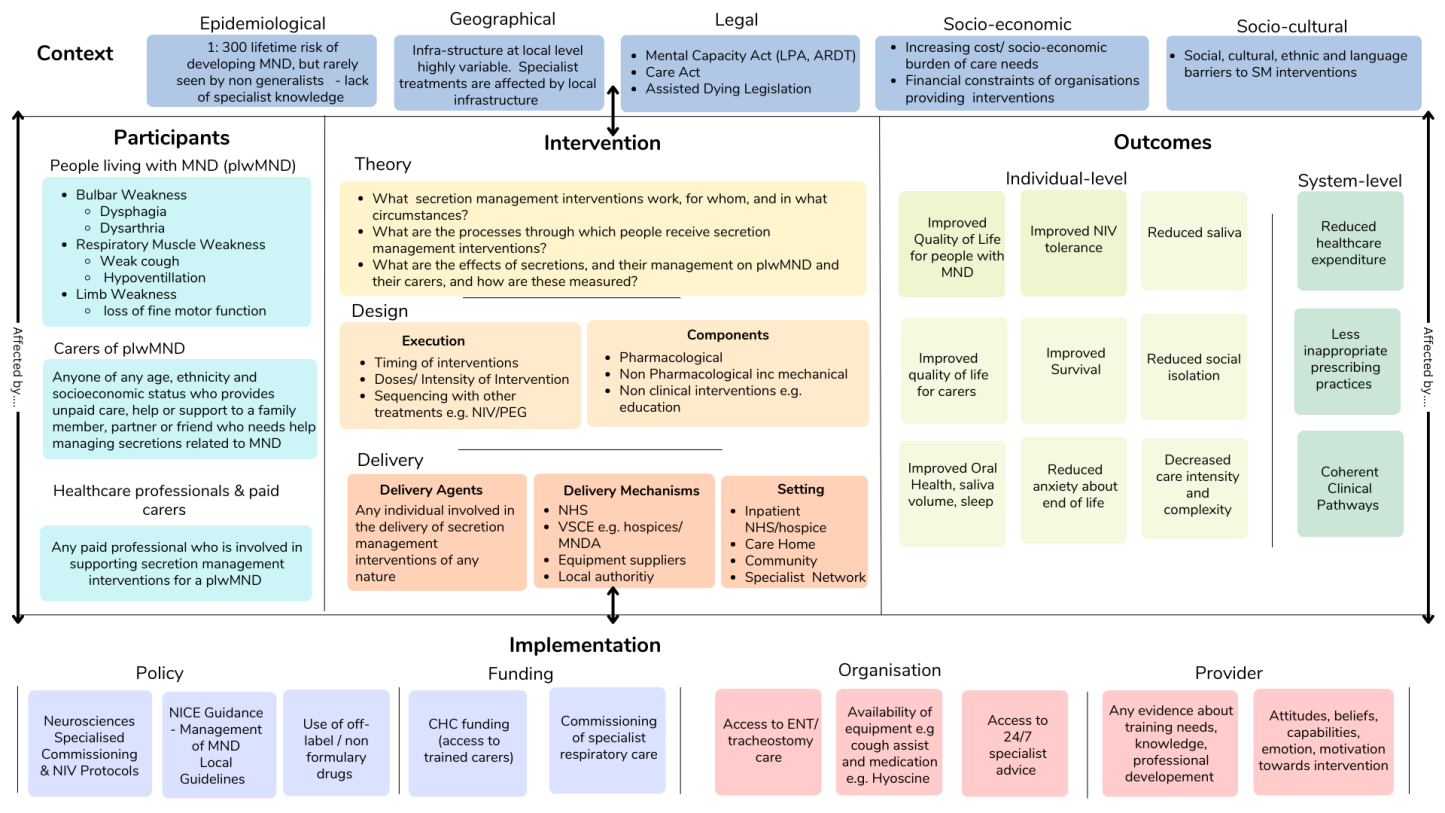
MND secretions interventions logic model. ADRT, Advance Decision to Refuse Treatment; CHC, Continuing Healthcare; ENT, Ear, Nose and Throat; LPA, Lasting Power of Attorney; MND, motor neuron disease; MNDA, Motor Neurone Disease Association; NHS, National Health Service; NICE, National Institute for Health and Care Excellence; NIV, Non-Invasive Ventilation; PEG, percutaneous endoscopic gastrostomy; SM, secretions management; VSCE, Voluntary, Community and Social Enterprise sector.

An initial logic model (figure 1) will be used to identify facets of complexity for the review questions.^9^ The initial logic model will act as a guide to give ‘an idea’ of the key interacting components of the intervention and will be adapted throughout the review process as new insights are identified from the literature. The initial logic model has been used to inform the target domains of the review, our PICOTS (Population, Intervention, Context, Outcomes, Timing and Setting) framework and has been shared with both MDT experts, and experts by experience to assist in conceptualisation and ensure a range of perspectives. The logic model will be revisited at the point of data extraction, data analysis and prior to dissemination, with clearly labelled versions of the model detailing how, and based on what information, changes have been made.

Given that this is an iterative review process, modifications to the protocol may be required as new and unexpected facets of complexity emerge.^9^ These will be recorded on PROSPERO and reported in the findings. For this reason, probable timelines for completion of the review are not possible at the outset, although approximately 8 months have been allowed for data extraction and synthesis.

## Methods

The review is designed in accordance with the principles, tools and guidance laid out in a series of articles published by the Agency for Healthcare Research and Quality on complex intervention systematic reviews.^7^

### Eligibility criteria

The ‘PICOTS’ framework (table 1) will be used to determine inclusion and exclusion criteria and to conceptualise the review. PICOTS adds ‘setting’ and ‘timing’ to the traditional PICO framework to map additional areas of complexity related to contextual factors.

**Table 1:** PICOTS categories

PICOTS category	
Population	People living with motor neuron disease Unpaid/family carers of people living with MND/ALS Professionals involved in the care of plwMND/ALS
Intervention/comparator	Any pharmacological or non-pharmacological interventions used in the management of secretions in MND/ALS (both respiratory and oropharyngeal secretions) Delivery of secretion management interventions in MND/ALS (delivery agents/mechanisms) Implementation of secretion management interventions in MND/ALS (funding/organisation/provider/policy)
Outcome	Any quantitative (eg, scores of validated instruments and/or other standardised measures) or qualitative (perceptions, thoughts, experiences) individual health and non-health outcomes, as follows: Health outcomes (individual): Person living with MND/ALS Physical and/or mental well-being Quality of life—non-invasive ventilation use Oral care Saliva/mucous Survival Carers of people living with MND/ALS Time or complexity of care needed Physical and/or mental well-being Quality of life Non-health outcomes (individual) Person living with MND/ALS Capacity for daily living Engagement Social relationships Sleep Confidence and competence in secretions management Carers of person living with MND/ALS Social networks/reduced social isolation Sleep Confidence and competence in secretions management Professionals involved in the care of people with MND/ALS Confidence and competence in secretions management Any systemic non-health outcomes including: Health services: Polypharmacy Unplanned attendance Recurrent attendance Service specifications Infrastructure requirements to deliver effective secretions management interventions
Timing	From diagnosis to death
Setting	All settings including the community, hospitals, care homes and hospices. The socioeconomic, geographical, legal, cultural and epidemiological context of intervention delivery will be considered.

MND/ALS, motor neuron disease/amyotrophic lateral sclerosis; plwMND/ALS, people living with MND/ALS.

The Intervention Complexity Assessment Tool for systematic reviews (iCAT_SR) has informed the development of inclusion criteria and search strategy. The iCAT SR is a tool to assess intervention complexity.^10^ The tool disaggregates constituent parts of an intervention, identifying relevant components and their delivery. Assessing secretion management interventions across a set of dimensions that categorise intervention complexity assisted in the development of our initial logic model. An initial rapid literature review was undertaken to populate an initial iCAT_SR table, which involves judgements as to level of intervention complexity over several ‘core’ and ‘optional’ domains (table 2). Information identified at the analysis stage may allow for judgements to be made with greater confidence and the table will be amended accordingly.

**Table 2:** ICAT_SR table: secretions management intervention complexity

	Judgement
Core dimension	
1. Active component included in the intervention	Moderate
2. Behaviours or actions of intervention recipients or participants to which the intervention is directed	Moderate
3. Organisational levels/categories targeted by the intervention	High
4. Level of tailoring	Moderate
5. Skill level required by those delivering intervention	Moderate
6. Skill level required by those receiving intervention	Varies
Optional dimension	
7. Degree of interaction between intervention components	High
8. Degree to which interventions are context dependent	Moderate
9. Degree to which interventions are modified by recipient factors	High
10. Nature of causal pathway between intervention and effect	n/a

### Information sources

#### Electronic sources

The following databases will be searched for English language studies:

Ovid Embase

EBSCO CINAHL

- EBSCO Academic Search Ultimate.
- Scopus.
- EBSCO PsycInfo.
- Ovid MEDLINE.
- Social Sciences Citation Index.

From 1996 to present. The cut-off date was selected as the multidisciplinary management of MND/ALS has evolved rapidly over that period, with older references less likely to be reflective of the complexity of managing secretions.^11^ Preliminary scoping identified very few relevant studies before this date. Searches will be supplemented by hand searching of grey literature, using the Public Health England Index of Grey Literature and Alternative Sources and Resources. While our searches will be restricted to studies published in English, if relevant contextual factors identified in our review require an international perspective selected non-English articles may be included.

#### Search strategy

The search strategy will aim to locate both published and unpublished studies using a three-step search strategy: Step 1 has been completed to inform this protocol. First, a preliminary scoping search of Ovid MEDLINE (PubMed) was undertaken to identify articles on the topic, with the assistance of an academic librarian. The text words contained in the titles and abstracts of relevant articles, and the index terms used to describe the articles were used to develop a full search strategy for reporting the name of the relevant databases/information sources (see table 3). The research team discussed and approved the list of key search terms. Next (Step 2), the search strategy, including all identified keywords and index terms, will be adapted for each included database and/or information source. Finally (Step 3), the reference lists of all included sources of evidence will be screened for additional studies (‘snowballing’). Database searches will be supplemented by hand searching of academic and grey literature.

**Table 3:** Example search strategy Ovid MEDLINE(R) ALL<1946 to 3 March 2025>

1	Sialorrhea/	1575
2	Saliva/	48 605
3	Salivation/	4151
4	Deglutition Disorders/	25 459
5	Sialorrhoea.tw.	144
6	Drool*.tw.	1787
7	Dribbl*.tw.	1238
8	Hypersalivat*.tw.	745
9	Dysphagi*.tw.	37 917
10	Swallow*.tw.	39 504
11	Dyspnea.tw.	50 839
12	Dyspnoea.tw.	13 468
13	Sialorrhea.tw.	824
14	Saliva*.tw.	127 097
15	Deglutition Disorder*.tw.	295
16	Respiratory Insufficiency/	36 991
17	(Airway adj2 (clear* or dysfunction* or block*)).tw.	3257
18	Respiratory muscle weakness.tw.	923
19	Cough*.tw. 71 291	
20	manual insufflation.tw.	12
21	(mechanical adj3 (insufflation or exsufflation)).tw.	274
22	frog breath*.tw.	5
23	glossopharyngeal breath*.tw.	65
24	breath stack*.tw.	54
25	air stack*.tw.	57
26	(assist* adj2 cough*).tw.	326
27	mucous.tw.	26 415
28	secretion*.tw.	409 615
29	Laryngospasm.tw.	1514
30	ventilat*.tw.	217 830
31	NIV.tw.	5085
32	respiratory insufficiency.tw.	8607
33	or/1–32	987 368
34	motor neuron disease.tw.	5258
35	MND.tw.	2466
36	Amyotrophic Lateral Sclerosis.tw.	32 079
37	ALS.tw.	50 389
38	Bulbar Palsy.tw.	563
39	Muscular Atrophy.tw.	10 687
40	kennedy* disease.tw.	380
41	or/34–40	74 842
42	and/33,41	4555

#### Study selection

Following the search, all identified citations will be collated and uploaded into EndNote 21/2023 (Clarivate Analytics, Pennsylvania, USA) and duplicates removed. Title and abstract screening then will be conducted by two independent reviewers using ASReview Lab (V.2.1), an open source machine learning tool for semi-automated citation screening. This software uses an active learning algorithm to prioritise the most relevant records for review. ASReview Lab will iteratively present records in order of predicted relevance, with reviewers making binary inclusion/exclusion decisions. The lead reviewer will screen until a predefined stopping criterion is reached. The stopping criteria will be a data-based strategy, where screening is stopped when the number of consecutive irrelevant papers exceeds 5% of the total dataset (eg, if there are 8000 abstracts, 400 abstracts have consecutively been rated irrelevant). The first 10% of the dataset will be screened by both reviewers to ensure that the eligibility criteria are being applied correctly. Excluded records will remain accessible for audit. A random sample of excluded records will be checked to ensure the model has not systematically excluded potentially eligible studies. Potentially relevant sources will be retrieved in full, and their citation details imported into the data management software Rayyan.^12^ The full text of selected citations will be assessed in detail against the inclusion criteria. Reasons for exclusion of sources of evidence at full text that do not meet the inclusion criteria will be recorded and reported. Any disagreements that arise between the reviewers at each stage of the selection process will be resolved through the use of a third reviewer. The results of the search and the study inclusion process will be reported in full in the final systematic review and presented in a PRISMA (Preferred Reporting Items for Systematic Reviews and Meta-Analyses) flow diagram.

### Data collection and analysis

Data will be extracted by two reviewers working independently. Study investigators will be contacted as required to obtain further information where it is unavailable or unclear in the original article.

### Assessment of risk bias

Two reviewers will independently assess the risk of bias in both quantitative and qualitative papers will be assessed using Gough’s Weight of Evidence framework.^13^ This will assess both study quality and relevance, a creation of weight of evidence framework that is both generic and review specific, and an overall judgement. Any disagreements in scoring will be decided by a third reviewer as needed.

### Data extraction and management

A visual map of the extracted information (using Excel) coded under different domains of the model will be constructed and used to explore and account for the mechanisms, processes and circumstances by which plwMND/ALS, their carers and care professionals use secretion management interventions. Management of the data will be through Rayyan and EndNote.

### Data analysis

The choice of analytical method depends on whether the existing evidence supports the use of a particular analytical approach. Due to the complexity of the topic, heterogeneity of evidence is anticipated, and it is unlikely to be possible to synthesise the evidence quantitatively to answer each review question.

If identified, studies that use qualitative methods for data collection and analysis will be analysed using the framework thematic synthesis approach. This method accommodates reports of complex interventions.

Where feasible, qualitative comparative analysis will be used to identify the combinations of intervention components, implementation features or contextual characteristics (eg, population, setting) that are associated with the intervention. Where possible, we will aim to identify hypotheses for subgroup analysis for future reviews of effectiveness, based on our findings. The review will report on how the analytical method used supported the aims of the review.

### Patient and public involvement and engagement and expert advisory group

Stakeholder engagement is recommended at all stages of development of a complex intervention review. The project will convene both professionals and experts by experience to guide the review throughout its stages. plwMND/ALS, family carers, care professionals, academics and policy leads (via the Motor Neurone Disease Association) have been involved in the development of the initial logic model.

Explicitly incorporating complexity into the topic scope and stakeholder discussions will prevent oversimplification of the topic area and review questions and ensure a shared understanding of the breadth and depth of review most helpful for plwMND/ALS, their family carers and care professionals supporting them.^9^

## Article summary and dissemination

### Ethical considerations

This is a systematic review that operates strictly with secondary source of data openly accessible in the public domain, therefore no ethical approval is required.

### Dissemination

The study findings will be disseminated in relevant academic outlets, practitioner and patient and public involvement and engagement fora. A lay summary (including a simplified logic model) will be produced to share in patient/carer forums such as support groups and newsletters. Where the findings of the review may be appropriate to inform clinical guidance, the lead investigator will work with relevant organisations to determine other appropriate routes of impact, such as patient information leaflets, existing quality standards and changes to existing clinical pathways.

### Review objectives

What secretion management interventions work, for whom and in what circumstances?

What are the mechanisms by which secretion management interventions are implemented by plwMND/ALS, their unpaid/family carers and care professionals?

What is the impact of secretions, and/or their management interventions on plwMND/ALS and their unpaid/family carers?
