# Supplementary material for: Understanding the complexity of living with, and managing, secretions in motor neuron disease/amyotrophic lateral sclerosis (MND/ALS/ALS): protocol for a complex intervention systematic review
Source: BMJ Open. 2025 Oct 10;15(10):e103704. doi: 10.1136/bmjopen-2025-103704 (PMC12517000; doi:10.1136/bmjopen-2025-103704)
Supplement: Supplementary file 1 [file bmjopen-2025-103704.reviewer_comments.pdf]

PEER REVIEW HISTORY

BMJ Open publishes all reviews undertaken for accepted manuscripts. Reviewers are asked to complete a checklist review form and are provided with free text boxes to elaborate on their assessment. These free text comments are reproduced below.

ARTICLE DETAILS

Title (Provisional)

Understanding the Complexity of Living with, and Managing, Secretions in Motor Neurone Disease/Amyotrophic Lateral Sclerosis (MND/ALS/ALS): Protocol for a Complex Intervention Systematic Review

Authors

Barry, Caroline; Farquhar, M; Hawkes, Matthew; Massey, Charlotte; Cross, Jane L.

VERSION 1 - REVIEW

**Reviewer**	**1**
**Name**	**Gonçalves, Filipe**
**Affiliation**	**University of A Coruna, Faculty of Health Sciences**
**Date**	**29-Jun-2025**
**COI**	**None**

The protocol is well-structured and addresses a highly relevant and challenging topic. The objectives are clearly defined, and the proposed methodology appears appropriate for achieving the intended outcomes. I would recommend only minor revisions to further enhance clarity and coherence:

- I would suggest using MND/ALS instead of just MND, for greater clarity and international alignment.

- Page 3, line 48: Please correct “oral dextromethorphan with quinine (DMQ)” to “dextromethorphan with quinidine (DMQ)”—they are chemically related diastereomers, but have different clinical applications.

- Page 5, Table 1 (lines 34–35): The phrase “living with” is missing “MND/ALS”—please revise.

- Page 9: “Table 2: PICOTS Categories” is repeated; this appears to be an error. Please correct it.

- Page 10, lines 56–58 (and similarly, page 11, lines 16–17): I would recommend limiting the resolution process to arbitration by a third reviewer only.

From my perspective, and based on the challenges I encounter in day-to-day clinical practice, as part of the review question, it would be important to assess adherence to the proposed therapies. In practice, even when targeted pharmacological interventions alter sialorrhea, they may modify its characteristics in ways that negatively affect swallowing, expectoration (rheology), and overall patient comfort—often leading to treatment discontinuation. This concern applies even to more sustained interventions such as botulinum toxin or radiotherapy. It is equally essential to evaluate potential systemic side effects and drug interactions that could limit therapeutic continuity.

**Reviewer**	**2**
**Name**	**Querin, Giorgia**
**Affiliation**	**University of Padova**
**Date**	**21-Jul-2025**
**COI**	**None**

This paper presents a well-designed, prospective, multicentre observational protocol aimed at systematically characterizing two under-investigated but highly disabling symptoms in patients with motor neuron disease (MND): sialorrhoea (excessive saliva) and thick mucus. These symptoms significantly affect patients' quality of life and may contribute to respiratory complications, yet they remain poorly quantified and understood in routine clinical practice.

The manuscript is clearly written, and the methodology is transparent and feasible.

The only really minor suggestion I would like to give to the authors is to indicate the probable timelines for the preparation of the review but I would otherwise accept it.

VERSION 1 - AUTHOR RESPONSE

Reviewer: 1
Dr. Filipe Gonçalves, University of A Coruna

Comments to the Author:
The protocol is well-structured and addresses a highly relevant and challenging topic. The objectives are clearly defined, and the proposed methodology appears appropriate for achieving the intended outcomes. I would recommend only minor revisions to further enhance clarity and coherence:

Thank you

- I would suggest using MND/ALS instead of just MND, for greater clarity and international alignment.

Amended
- Page 3, line 48: Please correct "oral dextromethorphan with quinine (DMQ)" to

“dextromethorphan with quinidine (DMQ)”—they are chemically related diastereomers, but have different clinical applications.

Amended

- Page 5, Table 1 (lines 34–35): The phrase “living with” is missing “MND/ALS”—please revise.

Amended

- Page 9: “Table 2: PICOTS Categories” is repeated; this appears to be an error. Please correct it.

Amended

- Page 10, lines 56–58 (and similarly, page 11, lines 16–17): I would recommend limiting the resolution process to arbitration by a third reviewer only.

Amended

From my perspective, and based on the challenges I encounter in day-to-day clinical practice, as part of the review question, it would be important to assess adherence to the proposed therapies. In practice, even when targeted pharmacological interventions alter sialorrhea, they may modify its characteristics in ways that negatively affect swallowing, expectoration (rheology), and overall patient comfort—often leading to treatment discontinuation. This concern applies even to more sustained interventions such as botulinum toxin or radiotherapy. It is equally essential to evaluate potential systemic side effects and drug interactions that could limit therapeutic continuity.

Many thanks for this comment- I agree. In the ICAT-SR table (page 7), the extent to which interventions used for sialorrhea (and respiratory secretions) may demonstrate complexity/variation is reflect in the optional dimensions points 7 – 9 . These domains consider the extent to which interventions may interact with each other (e.g. the co-prescription of mucolytics and anti-muscarinic), degree to which interventions are context dependent and the extent to which interventions are modified by recipient factors (e.g. adherence). You can see from these table that I have rated the complexity of both recipient factors, and intervention interaction as “high” in recognition of the point you are making. The logic model which results from the review will further expand on the relationship between context, mechanism and action of these interventions.

Reviewer: 2
Dr. Giorgia Querin, University of Padova

This paper presents a well-designed, prospective, multicentre observational protocol aimed at systematically characterizing two under-investigated but highly disabling symptoms in patients with motor neuron disease (MND): sialorrhoea (excessive saliva) and thick mucus. These symptoms significantly affect patients’ quality of life and may contribute to respiratory complications, yet they remain poorly quantified and understood in routine clinical practice. The manuscript is clearly written, and the methodology is transparent and feasible.

Many thanks for this feedback.

The only really minor suggestion I would like to give to the authors is to indicate the probable timelines for the preparation of the review but I would otherwise accept it.

Many thanks for this. We hope to complete the review within 8 months, but, in comparison to a standard systematic review, a complex intervention systematic review is an iterative process therefore it is harder to give definitive timescales for completion. This is described in the methodology section on page 4, reference Kelly, 2017. I have added a sentence within the Methodology > approach section to this effect if you think it would be helpful to include this.

We trust that the revisions are acceptable to our reviewers. Please note, that a change to the screening process was made between submission of the manuscript, and the reviewers' comments. This has been amended on the new manuscript, to reflect the use of a semi-automated machine learning tool in the screening process.

Thank you for your time and consideration,
